# Social administration of juvenile hormone to larvae increases body size and nutritional needs for pupation

**DOI:** 10.1098/rsos.231471

**Published:** 2023-12-20

**Authors:** Matteo A. Negroni, Adria C. LeBoeuf

**Affiliations:** ^1^ Department of Biology, University of Fribourg, Chemin du Musée 10, 1700, Fribourg, Switzerland; ^2^ Department of Zoology, University of Cambridge, Downing Street, Cambridge, UK

**Keywords:** social regulation of development, social insects, handfeeding larva, social transfer, trophallaxis, juvenile hormone

## Abstract

Social insects often display extreme variation in body size and morphology within the same colony. In many species, adult morphology is socially regulated by workers during larval development. While larval nutrition may play a role in this regulation, it is often difficult to identify precisely what larvae receive from rearing workers, especially when larvae are fed through social regurgitation. Across insects, juvenile hormone is a major regulator of development. In the ant *Camponotus floridanus*, this hormone is present in the socially regurgitated fluid of workers. We investigated the role the social transfer of juvenile hormone in the social regulation of development. To do this, we administered an artificial regurgitate to larvae through a newly developed handfeeding method that was or was not supplemented with juvenile hormone. Orally administered juvenile hormone increased the nutritional needs of larvae, allowing them to reach a larger size at pupation. Instead of causing them to grow faster, the juvenile hormone treatment extended larval developmental time, allowing them to accumulate resources over a longer period. Handfeeding ant larvae with juvenile hormone resulted in larger adult workers after metamorphosis, suggesting a role for socially transferred juvenile hormone in the colony-level regulation of worker size over colony maturation.

## 1. Introduction

The regulation of development of multicellular organisms generally comes about through coordination and molecular communication between cells and tissues [1], but can also be controlled socially through nutrition, pheromones or through the social transfer of bioactive molecules from parents or conspecifics to the young [2–7]. Socially transferred materials can strongly impact size, morphology and fitness of the resulting adults [2,5–7]. Social insect species count among the best examples of intra-specific variation in body size, with multiple morphological castes, including minor workers, major workers and queens [8–10]. While genetics can influence adult morphology and body size, caste determination in social insects is often strongly dependent on environmental factors, including socially manipulated environmental features such as temperature, nutrition and colony size [11–13]. Indeed, in many species of termites, ants, bees and wasps, morphological variation is observed within the same genotype, providing an ideal system to study mechanisms underlying social, epigenetic and developmental regulation of size. Despite many decades of study, the social and molecular mechanisms that govern the developmental trajectories of the different castes are not fully understood [14–16].

Variation in larval nutrition clearly plays an important role in caste determination and the regulation of adult morphology [17,18]. For example, in the honeybee *Apis mellifera* early-stage bipotent larvae fed with royal jelly or worker jelly develop into larger fertile queens or smaller non-reproductive workers, respectively [17,19]. Proteins that make up these jellies are synthesized by nurse workers who feed the larvae [19] and adapt quantitatively and qualitatively the composition of their jelly diet [17,20]. Although there is evidence for a role for nutrition in caste determination in social insects other than in honeybees [11,12,18,21–24], the molecular and social mechanisms of caste fate determination and adult body size are far less understood.

Combined with nutrition, developmental time is an additional parameter that can influence adult size and morphology, as it can impact the total amount of resources that can be accumulated during the larval phase. Under a similar diet and feeding frequency, larvae that have an extended developmental time may be able to accumulate more resources than faster developing larvae [25,26].

In insects, developmental time is essentially controlled by the most well-known insect hormone, juvenile hormone [27]. Juvenile hormone is a lipophilic sesquiterpenoid hormone produced in the corpora allata, endocrine glands situated near the brain [27,28]. There are several juvenile hormones in insects, and their effects on adult and larval physiology are diverse, covering reproductive, immune, growth and stress-resistance functions [29]. As shown in solitary species, juvenile hormone delays moulting and pupation, and combined with sufficient nutrition, leads to larger adult body size [30]. Various studies have shown that juvenile hormone III, the most common form of juvenile hormone in social Hymenoptera [31], affects adult size and morphology in ants [7,22,27,32,33]. In the ant *Harpegnathos saltator,* treating larvae with juvenile hormone increases their likelihood of developing into larger adults or into queens [33]. Juvenile hormone is thus a good candidate for the regulation of body size and caste determination through its effect on larval development or nutritional needs for pupation in social insects [34,35]. Whether the regulation of body size through juvenile hormone comes about through manipulation by rearing workers or by variation in the synthesis or degradation of juvenile hormone by the larva itself, has yet to be established [7,36,37].

In numerous species of ants, wasps and bees, larvae are fed by workers through a social transfer of regurgitate stored in the crop (trophallaxis) [12,36,38,39]. Yet, there have been few studies on social regulation of larval development through the social transfer of worker-derived components beyond the major royal jelly proteins in *A. mellifera*. The reason for this is that it is often difficult to know exactly what larvae receive from workers in these one-to-one interactions [40]. In the ant *Camponotus floridanus*, the worker size distribution is bimodal with major and minor workers differing in size and in allometry [24,41]; in this species, the transfer of nutrients in the colony is done almost exclusively by trophallaxis [36,37,40]. Developmental mechanisms modulating allometric differences between major and minor workers in *C. floridanus* are still not established but have been suggested to involve juvenile hormone [41]. In this species, analysis of social regurgitate revealed the presence of many worker-derived (endogenous) components in high abundance including proteins, RNA and hormones, in addition to exogenous material [36,37,40]. That juvenile hormone counted among the crop regurgitate components suggests an external regulation of larval growth and developmental time by the rearing workers [7,24]. In 2016 and in 2018, LeBoeuf *et al*. [36,37] tested the effect of social administration of juvenile hormone on *C. floridanus* larval development by supplementing the diet of rearing workers with juvenile hormone III. While this treatment did not impact the allometry, it increased the head width of resulting adults and the rate of development to metamorphosis. However, this experimental design could not distinguish if juvenile hormone supplementation had an impact on larvae through effects on worker rearing or through a direct effect on the larvae themselves. Moreover, the precise amount of juvenile hormone transferred socially to the larvae could not be regulated.

Here, we tested the effect of orally administered juvenile hormone on larval development, growth, pupation rate and on the resulting adult size and morphology in the ant *C. floridanus*. To do this, we developed a handfeeding method to provide developing larvae with precise quantities of juvenile hormone directly. We expected this juvenile hormone supplementation to positively affect larval growth and the resulting adult body size, possibly through modulation of developmental time, larval nutritional needs for pupation or larval begging behaviour. As juvenile hormone impacts the physiological transitions of molting and growth, we also expected an impact on larval metabolic rate [42,43]. Finally, if the social transfer of juvenile hormone from worker to larva is necessary to ensure a proper development, we expected juvenile hormone to have a positive effect on larval survival rate through pupation.

## 2. Results

**Figure 1.**
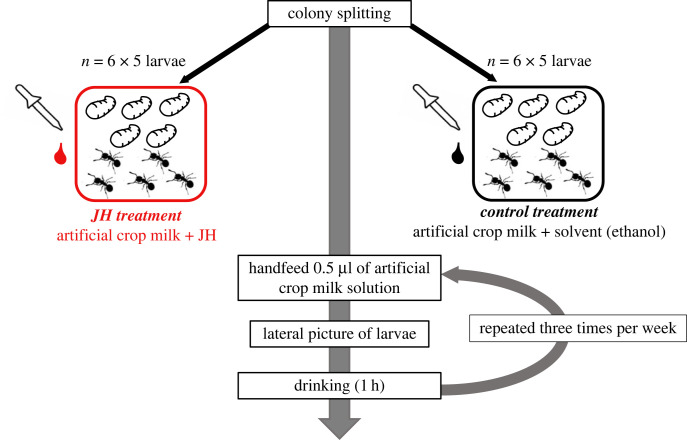
Illustration of experimental design. Twelve experimental nests, composed of five larvae and five workers, were created from six source colonies, and shared equally between the juvenile hormone (JH) treatment (in red) and the control treatment (in black). Larvae were handfed three times per week with 0.5 µl of artificial crop regurgitate containing juvenile hormone or solvent until pupation or death of larvae. Before feeding sessions, larvae were isolated from their workers, photographed laterally, fed and then put back with workers.

In order to test the influence of juvenile hormone on larval physiology, behaviour and development, we developed a handfeeding protocol that allowed us to precisely control the feeding of ant larvae. With this method, larvae are provided with a precise volume of food three times per week. Outside of their feeding, larvae were cared for by nursing workers. Our handfeeding protocol consisted in first isolating larvae from their workers and placing them on their backs into larva-sized wells made in a Play-Doh plate. Then, using a Hamilton syringe, we placed a droplet of artificial crop regurgitate solution on the mouthparts of each larva, while using a needle to keep the neck extended. They were left to feed for an hour after which each larva was cleaned with a moistened cotton-tipped toothpick before being returned to their respective workers. While larvae were fed, workers were also fed—simultaneous satiety of workers and larvae minimized any social fluid transfer from adults to larvae or from larvae to adults. Worker diet contained fluorescence and larval diet contained blue dye such that larval intake of either food could be monitored. We handfed a group of 30 larvae with a mixture of artificial crop regurgitate with juvenile hormone, and another group of 30 larvae with artificial crop regurgitate and solvent alone. These treatments were conducted until pupation or death of the larvae (figure 1).

**Figure 2.**
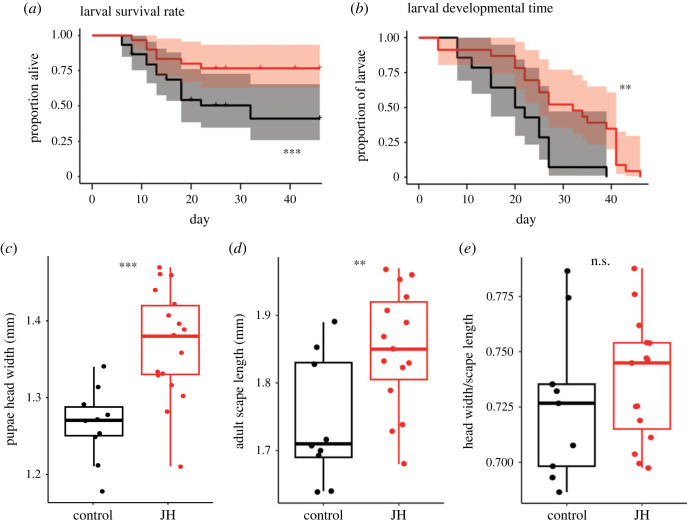
Juvenile hormone (JH) feeding of larvae increases survival, developmental time and impacts morphology of the resulting adults. (*a*) Larval survival over time; (*b*) proportion of larvae that have not pupated over time among the larvae that survived until pupation; boxplots of morphological measurement according to the treatment for (*c*) head width, (*d*) scape length, and (*e*) the ratio between head width to scape length. Juvenile hormone treatment figures in red and control treatment in black. The survival of larvae differed between treatments (survival mixed model: *p* = 5.6 × 10^−6^) as did the developmental time before pupation (survival mixed model: *p* = 2.1 × 10^−3^). Differences between treatments for head width LMM: *p* = 2.0 × 10^−5^; for scape length LMM: *p* = 3.3 × 10^−3^; head width/scape length LMM: *p* = 0.34 (full statistical results presented in table 1).

As expected, larval survival was much higher in the juvenile hormone treatment compared to the control (survival mixed model: *p* = 2.2 × 10^−6^; table 1 and figure 2*a*). In the control treatment 14 larvae reached pupation (46.7% of total larvae), 10 of these pupae reached metamorphosis (33.3% of total larvae) and nine emerged as adults. In the juvenile hormone treatment, 23 larvae reached pupation (76.7% of total larvae), 17 underwent metamorphosis (56.7% of total larvae) and 15 emerged as adults. Analysing the time needed to reach pupation revealed that handfeeding juvenile hormone to larvae extended their developmental time (survival mixed model: *p* = 2.1 × 10^−3^; table 1 and figure 2*b*). Next, to look at the influence of our handfeeding treatment on resulting adult morphology, the measurement of pupae revealed a positive effect of juvenile hormone on resultant adult head width (linear mixed model (LMM): *p* = 2.0 × 10^−5^; table 1 and figure 2*c*) and scape length (LMM: *p* = 3.3 × 10^−3^; table 1 and figure 2*d*), with an average of 1.37 and 1.85 mm for head width and scape length, respectively, in the juvenile hormone treatment and 1.27 and 1.74 mm for head width and scape length, respectively, in the control treatment. However, no difference in the ratio between these two variables (head width/scape length) was detected between treatments (LMM: *p* = 0.34; figure 2*e*), revealing an absence of impact of juvenile hormone treatment on the allometry of the adults.

**Table 1.** Statistical results of the effects of treatment on survival, time until pupation, head width, scape length and the ratio between the two last, and the last larva mass record according to the conservative algorithm. (Here we present the results from the ANOVA of the models, specifying the type of mixed model used, all of them implemented with colony identity and fragment identity as random factors.)

type of model	response variable	*χ*^2^	d.f.	*p*
survival mixed model	time until death	22.38	1	2.2 × 10^−6^
	time until pupation	9.45	1	2.1 × 10^−3^
linear mixed model	head width	18.22	1	2.0 × 10^−5^
	scape length	8.64	1	3.3 × 10^−3^
	head width/scape length	0.92	1	0.34
	last mass record	11.63	1	6.5 × 10^−4^

To investigate mechanisms underlying the positive effect of juvenile hormone treatment on adult scape length and head width, we looked at larval body mass in relation to time, and with handfeeding treatment. There are no available methods to safely tag larvae, so to monitor larval identity over weekly larval mass measurements, we used two different algorithms, one ‘restrictive’ and one 'conservative'. In the restrictive algorithm, we assumed: (i) consistency in the ordering of the larvae according to their mass; (ii) if one larva pupates, it is the larva that was largest the previous week; and (iii) if there was a death, it was the smallest larva of the previous week. In the conservative algorithm, larval identity across weekly measurements, pupations and deaths were randomly assigned without the assumption that larval weight must increase over time; the only constraints were a data-informed size threshold that had to be reached before pupation and a data-informed maximal percentage increase in larval weight between weeks. The conservative algorithm was used 10 times to yield 10 simulated datasets and *p*-values were averaged for the statistics performed. For clarity, we present results arising from the restrictive algorithm though results from the 10 simulations can be found in the electronic supplementary material.

**Figure 3.**
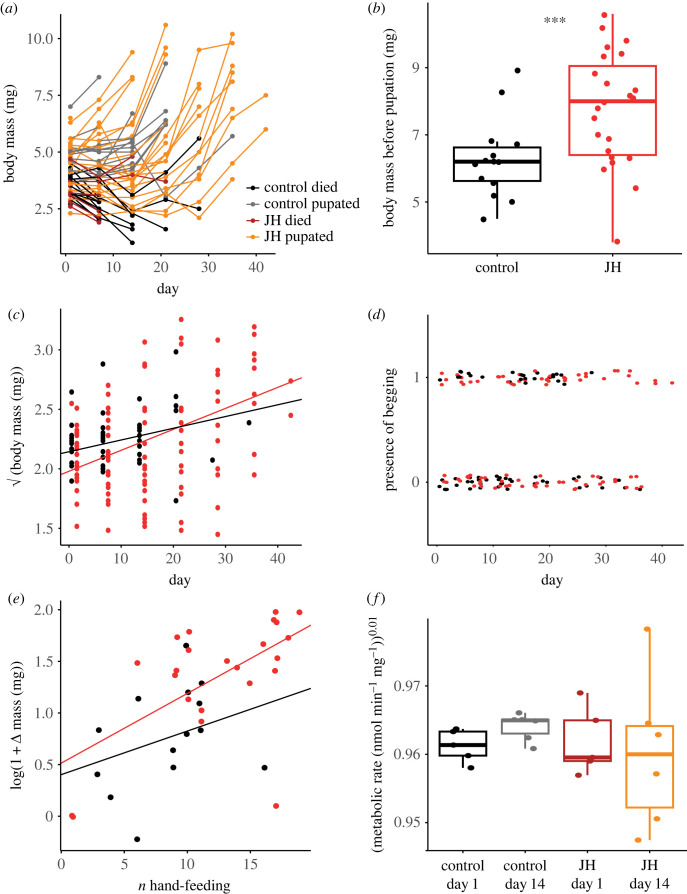
Effect of handfeeding treatments on larval body mass over time until pupation and on metabolic rate. On the top line of the figure: (*a*) individual larval mass over time, with juvenile-hormone (JH)-treated larvae that reached pupation in red and those that died before the end of the experiment in orange, and larvae from the control treatment that reached pupation in grey and those that died before the end of the experiment in black; (*b*) boxplots of the last individual larval mass record according to treatment for the restrictive algorithm (red: juvenile hormone treatment; black: control treatment; significant differences between treatments: LMM: *p* = 6.5 × 10^−4^; ***p<0.001). On the middle line of the figure: plots of individual mass over time (*c*) and larval begging over time (*d*) in interaction with treatment. There is a positive effect of time on larvae body mass (LMM: *p* = 9.19 × 10^−11^) with no effect of treatment (LMM: *p* = 0.38); nor the interaction between these variables (LMM: *p* = 0.22). There is no effect of time on the presence of larval begging behaviour (generalized linear mixed model (GLMM) binomial: *p* = 0.84) with no effect of treatment (GLMM binomial: *p* = 0.35) nor of the interaction between these variables (GLMM binomial: *p* = 0.91). On the bottom line of the figure: (*e*) influence of treatment (LMM: *p* = 4.8 × 10^−2^), the number of handfeeding events (LMM: *p* = 5.40 × 10^−4^) and their interaction (LMM: *p* = 0.55) on larval change in mass; (*f*) change in metabolic rate according to the stage of the experiment (LMM: *p* = 0.98), according to treatments (LMM: *p* = 0.35) and their interaction (LMM: *p* = 0.30 The full statistical results are presented in tables 1 and 2.

Analysing larval growth over time (figure 3*a*; electronic supplementary material, figure S1), we observed that the juvenile-hormone-treated larvae grew bigger than did control-treated larvae, in line with our expectations. While the initial larval masses across treatments were equivalent (LMM: *χ*^2^_1_= 5.46 × 10^−2^, *p* = 0.84), the comparison of the last mass record before pupation between treatments revealed that juvenile-hormone-treated larvae were heavier than controls, according to both algorithms (restrictive algorithm, LMM: *p* = 6.5 × 10^−4^; table 1, figure 3*b*; conservative algorithm, LMMs: average *p* = 3.64 × 10^−2^, median *p* = 2.4 × 10^−2^; electronic supplementary material, table S1 and figure S2). However, these differences in final larval body size did not result from differences in growth rate as the analysis of individual larval body mass over time and according to treatment showed no interaction between these two variables (restrictive algorithm, LMM: *p* = 0.22; figure 3*c*; conservative algorithm LMMs: average *p* = 0.62, median *p* = 0.70; electronic supplementary material, table S2 and figure S3), nor an effect of treatment (restrictive algorithm: *p* = 0.38; figure 3*c*; conservative algorithm, LMMs: average *p* = 0.77 median *p* = 0.80; electronic supplementary material, table S2 and figure S3) but only an effect of time (restrictive algorithm, LMM : *p* = 9.19 × 10^−11^; figure 3*c*; conservative algorithm, LMMs: average *p* = 1.18 × 10^−10^, median *p* = 7.84 × 10^−11^; electronic supplementary material, table S2 and figure S3).

**Table 2.** Statistical results of the effects of treatment, time and their interaction on larval mass (i) using the restrictive algorithm, (ii) larval begging behaviour and (iii) larval metabolic rate. (These analyses were done on models implemented with colony identity as random factors and models (i) and (ii) with fragment identity as additional random factor, models (i) and (iii) were linear mixed models, while model (ii) was a binomial generalized mixed model. In contrast with the two other models, in model (iii), the variable time is discretized in two levels: initial and day 14.)

explanatory variables	(i) √(larval mass)			(ii) presence of larval begging			(iii) (larval metabolic rate)^0.1^		
	*χ*^2^	d.f.	*p*	*χ*^2^	d.f.	*p*	*χ*^2^	d.f.	*p*
time	41.98	1	9.19 × 10^−11^	3.90 × 10^−2^	1	0.84	5.0 × 10^−4^	1	0.98
treatment	0.76	1	0.38	0.88	1	0.35	0.88	1	0.35
time × treatment	1.44	1	0.22	1.3 × 10^−2^	1	0.91	1.1	1	0.30

**Figure 4.**
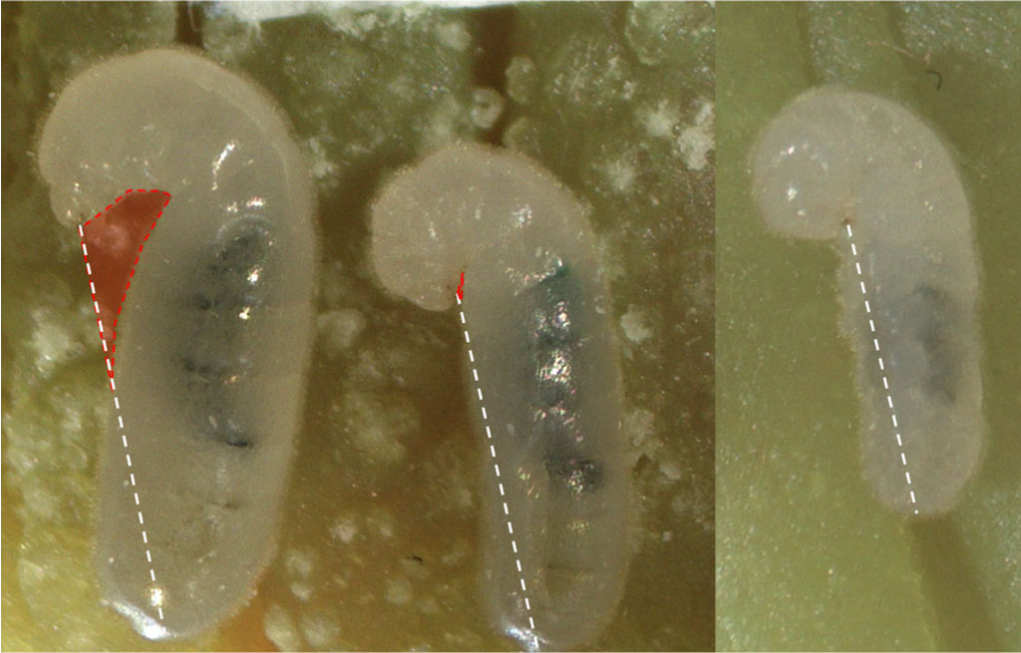
Lateral picture of larvae for begging behavioural measurements. The quantification of begging behaviour was done by measuring the area (transparent red) between the mandibles, the neck, the rest of the body and the imaginary line (white dashed) linking the mandibles and the posterior extremity of the larva, standardized by the total area of the larva. According to this measurement the larva on the left begs more than the larva in the middle and larva on the right is not begging.

To understand whether juvenile-hormone-related differences in size and pupation came about owing to differential treatment by workers, we wanted to measure larval begging behaviour. Begging, through the extension of the larval mouthparts away from the body, generally allows larvae to modulate the amount of food they receive from workers [44]. For a larva to be fed through mouth-to-mouth trophallaxis, their mouthparts should be physically accessible to their workers feeding them. Therefore, based on this and previous work on larval begging [44], we measured the space between the mouthparts and the rest of the body as proxy of larval begging, and we analysed the presence of begging larva (the presence of space between mouthparts and ventral part of the neck) in response to oral juvenile hormone supplementation (figure 4). We found no effect of this treatment on the presence of larval begging (LMM: *p* = 0.35; table 2 and figure 3*d*), and there was no effect of time (LMM: *p* = 0.84; table 2 and figure 3*d*) nor interaction between time and treatment (LMM: *p* = 0.91; table 2 and figure 3*d*) on larval begging likelihood. Consistent with this observation, the analysis of larval fluorescence (Wilcoxon pairwise rank test, with Bonferroni correction, between control and juvenile hormone treatment: *p* = 0.83; electronic supplementary material, figure S4) did not show significant differences between treatments suggesting that juvenile hormone does not cause workers to feed larvae more frequently. These observations all together suggest that the differences in mass at pupation do not result from a change in feeding frequency from workers.

To unravel the link between developmental time and body size, we found a larger change in larval mass over the experiment in the juvenile hormone treatment compared to the control, but only with the restrictive algorithm (LMM: *χ*^2^_1_ = 3.97, *p* = 4.80 × 10^−2^; figure 3*e*; conservative algorithm average *p* = 0.44, median *p* = 0.45; electronic supplementary material, table S3, figure S5). We also found a positive association between the individual increase in larval body mass and the number of feeding events that a given larva had sustained (restrictive algorithm LMM: *χ*^2^_1_ = 11.96, *p* = 5.4 × 10^−4^; figure 3*e*; conservative algorithm average *p* = 3.7 × 10^−2^, median *p* = 1.3 × 10^−2^; electronic supplementary material, table S3 and figure S5), but this effect was independent of treatment (restrictive algorithm LMM: *χ*^2^_1_ = 0.35, *p* = 0.55; figure 3*e*; conservative algorithm average *p* = 0.46, median *p* = 0.36; electronic supplementary material, table S3 and figure S5), indicating that generally, the longer the developmental time at a constant feeding frequency, the more growth occurs. These results suggest that the absolute amount of nutrients accumulated through development positively impacts larval growth. Finally, testing for physiological changes related to juvenile hormone supplementation, we found no change in the average-larval metabolic rate per fragment over time (LMM: *p* = 0.98; table 2 and figure 3*f*) nor an effect of the treatment (LMM: *p* = 0.35; table 2 and figure 3*f*) and no significant interaction between these factors (LMM: *p* = 0.30; table 2 and figure 3*f*).

## 3. Discussion

We investigated the role of the social transfer of juvenile hormone in the social regulation of development in *C. floridanus*. We socially administered artificial crop regurgitate to larvae that was supplemented or not with juvenile hormone through a newly developed handfeeding method. Juvenile hormone extended larval developmental time and increased larval mass at pupation resulting in larger adult workers. These effects did not result from changes in feeding behaviour by the rearing workers or through larval begging. Instead, juvenile hormone extended larval development, which allowed them to accumulate resources for a longer period and reach larger size at pupation. Finally, our results indicate that larvae rely on socially transferred juvenile hormone from workers to complete their development, suggesting homosynergetic metabolic division of labour between workers and larvae [39].

Among the effects of our handfeeding treatments on larval development, we found that juvenile hormone increased the final larval body mass before pupation, which was verified with both of our algorithms used to impute larval identity. Larval growth requires nutrient intake [45,46] and therefore an increased final larval mass is expected to result from a higher absolute amount of nutrients accumulated during development. Our results suggest that the increase in final larval mass did not result from a change in feeding frequency by the workers. Indeed, the analysis of fluorescence did not show differences between the amounts of adult feeding between treatments, and based on our assessment of begging, our juvenile-hormone-treated larvae did not ask more for food from their workers either. Interestingly, we found that the change in larval mass was positively associated with the number of handfeeding events experienced by larvae and this association was independent of treatment. These results first indicate that the accumulation of nutrients during developmental time dictates larval growth, and second, that the effect of the number of handfeeding events on growth was independent from treatment, suggesting that the efficiency of converting nutrients into body mass was not affected by juvenile hormone [46]. Together these results suggest that for juvenile-hormone-treated larvae, to reach a larger size at pupation, larvae must accumulate more nutrients during development through an extension of developmental time, which would maximize the number of handfeeding events experienced under this constant feeding regime [20,21,24,46,47]. Supporting this hypothesis, our analyses of growth rate and developmental time revealed that juvenile hormone-treated larvae, instead of growing faster, grew for a longer period compared to the control-treated larvae. Biologically this means that socially administered juvenile hormone increases the nutritional needs of larvae, extending the developmental phase and allowing them to reach a larger size at pupation [46].

That juvenile hormone-treated larvae pupated at a larger size than control-treated larvae while keeping the growth rate unchanged stands in contrast with what is observed in honeybees, where the larger larval size of queen-destined larvae before metamorphosis comes about through faster growth [17]. Instead of a change in growth rate, in our experiment it seems that larval size at pupation instead depends on a juvenile-hormone-mediated modulation of developmental time. A link between developmental time and adult body size has been shown in *Myrmica rubra* where fast-developing summer brood produce smaller pupae than slow-developing winter brood [47]. A positive effect of juvenile hormone at the larval stage on the resulting adult body size has been observed in *H. saltator* where an artificial elevation of juvenile hormone (with methoprene, a non-hydrolysable juvenile hormone analogue) in third- and fourth-instar larvae increased the likelihood of larvae to develop into queens, larger than workers, through a delay of metamorphosis [33]. In *C. floridanus* whether major-, minor- and queen-destined larvae differ in their developmental times remains to be investigated.

Our morphological analysis of pupae revealed a positive effect of our juvenile hormone supplementation treatment on head width, suggesting that the developmental regulation of this trait involves the social transfer of juvenile hormone. The worker head width distribution is bimodal in *C. floridanus* colonies and is the trait that best discriminates minors from majors [41]. In our experiment, larvae supplemented with juvenile hormone tended to develop into more major-like adult workers, which could implicate juvenile hormone in caste determination as is the case in *Pheidole pallidula* ants [7,24]. However, the largest head width observed in our juvenile hormone treatment was smaller than the smallest head width observed among majors in Alvaro *et al.* [41]. Moreover, minors and majors are characterized by differences in the ratio between head width and scape length. Here we show that juvenile hormone affected scape length in the same direction as head width but did not affect the ratio between these two traits, suggesting that our artificial oral administration of juvenile hormone was not sufficient to drive the developmental trajectory toward majors [41].

Demonstrating that social transfer of juvenile hormone is a mechanism for social regulation of larval development and adult morphology in *C. floridanus*, would require showing that workers alter the amount of juvenile hormone administered to larvae according to colony needs. For example, workers are generally larger in mature colonies compared to smaller founding nests [48,49]. If juvenile hormone is involved in this switch in the type of newly produced workers over colony maturation, we would expect juvenile hormone concentration in the crop regurgitate to also increase with colony size and maturity. Further experiments are required to test this prediction.

As in LeBoeuf *et al.* [36], the survival rate of juvenile hormone supplemented larvae was much higher compared with the control. Juvenile hormone is essential in larval development in insects [50]; for example, when juvenile hormone methyltransferase is knocked down in potato beetles, larval survival reduces dramatically [51]. However, in contrast to solitary species, *C. floridanus* larvae may receive juvenile hormone from their workers, and therefore a certain concentration of juvenile hormone in their social regurgitate diet may be necessary for their development through metamorphosis. Therefore, the transfer of worker-derived juvenile hormone through feeding may be important for their development. If so, this would be an example of homosynergetic division of metabolic labour between workers and larvae [39]. Through evolution, this division of metabolic labour may have resulted in larvae being developmentally dependent on their workers for a sufficient synthesis of juvenile hormone [39]. Assessing the economical aspect of this division of labour requires measuring the physiological costs of synthesis of juvenile hormone as well as benefits of receiving it socially. Furthermore, it has been recently shown that *C. floridanus* have evolved a form of juvenile hormone esterase in the blood brain barrier [52] that protects individual's brains from juvenile hormone which might otherwise affect their behaviour. Potentially this enzyme might represent an adaptation to ensure worker behavioural integrity, despite the high titre of juvenile hormone that must be produced to be fed to larvae. Finally, a developmental dependency of larvae on workers could be a colony-level mechanism for ensuring a social control of larval development and reducing conflict between larvae and the rest of the colony regarding their developmental trajectory [53].

Bringing larvae through metamorphosis using our handfeeding technique was successful for up to 56.7% of the larvae. To the best of our knowledge, this is the first time that larvae have been artificially reared through handfeeding in an ant species where larvae are fed exclusively by trophallaxis from adults. The method we have developed opens many doors to investigate the impacts of specific molecules (socially transmitted or not) on larval development in ants beyond *C. floridanus* [38].

Beyond the larvae that survived, we noticed major losses occurring through cannibalism and developmental issues between pupation and metamorphosis. Similarly high rates of larval cannibalism have been observed in *C. floridanus* even in the absence of starvation conditions, suggesting that the cannibalized larvae may partly be not viable but rather are ‘destined’ to be recycled by the workers [39,54].

Contradicting our expectations, metabolic rate did not change during larval development nor differed between treatments, with no significant interaction between these parameters. In insects, juvenile hormone is known to induce an increase in metabolic rate as shown both at the adult and larval stage [43,55,56]. Thus, our results contrast with previous literature but given our sample sizes for metabolic rate measurements and the numerous differences between social and solitary insects make the interpretation of this result delicate. In the solitary insect species *Leucophaea mederae,* an analogue of juvenile hormone simulates the synthesis of yolk proteins in larvae [57]. In *C. floridanus,* yolk proteins such as vitellogenin are found in the social regurgitate of workers, possibly underlying metabolic division of labour between larvae and workers, where these proteins are produced by workers but used by larvae [39,40]. Such metabolic division of labour may explain why juvenile hormone can impact larval growth while larval metabolic rate remained unchanged—because the materials required for growth are transferred to the larvae by workers, so the larvae do not need to perform costly metabolism to grow.

To conclude, our results reveal that the artificial oral administration of juvenile hormone in *C. floridanus* larvae increases larval nutritional needs, which translates into an extension of developmental time, and in turn, an increase in adult worker body size. While our findings suggest that juvenile hormone is involved in the social regulation of larval development, whether workers can adapt the amount of juvenile hormone transferred to the larvae according to colony needs, remains to be investigated. Unexpectedly juvenile hormone seems not to affect worker allometry. Regarding the huge diversity of molecules and proteins present in the crop regurgitate of *C. floridanus*, the role of juvenile hormone on larval development should be treated in combination with other components of this rich fluid that may interact together. Finally, this study describes a new method of larval handfeeding mimicking trophallaxis, and as such, it opens the doors for further experiments manipulating the larval diet with various components from the crop regurgitate and brings an opportunity for investigating mechanisms involved in the social regulation of larval development in other species.

## 4. Material and methods

### 4.1. Colony collection and maintenance

We used a total of *n* = 6 queen-right colonies of *C. floridanus* collected between 2019 and 2020 and with colony sizes ranging between 130 and 320 workers (mean = 205; s.e. ± 68.34; table 3). Colonies were kept in cylindrical plastic boxes of 32 cm diameter with artificial nests made of one or two glass tubes, each filled partially with water, closed with a cotton ball, and covered with a red transparent foil. Colonies were maintained in controlled conditions at (25°C; 60% humidity; 12 : 12 h day/night cycle) and fed once per week with artificial Bhatkar-diet [58], crickets and provided with water and 15% sugar water ad libitum.

**Table 3.** Colony information.

colony identity	collection date	site	worker number
C1	28 Feb 2020	Conch Key	180
C2	20 Jan 2019	Fiesta Key	220
C3	21 Jan 2019	Fiesta Key	250
C4	19 Jan 2019	Sugarloaf Key	230
C5	18 Jan 2019	Craig Key	320
C6	21 Jan 2019	Fiesta Key	130

### 4.2. Experimental design

First, we measured the protein and sugar concentration of crop regurgitate collected from our six colonies. To do so, we collected between 6.5 and 11 µl of crop regurgitate from each colony by anesthetizing randomly picked nurse ants with CO_2_ and gently squeezing the abdomen while collecting the fluid exuded from the mouth using a glass capillary. We then analysed protein concentration, using the Qbit 4 fluorometer (with the broad range protein assay kit Qubit Protein BR Assay, from ThermoFisher Scientific), and sugar concentration using the refractometer (refractometer RBR32-ATC), from a pool of each colony's crop content. These measures resulted in an average protein concentration of 37 mg ml^−1^ and an average sugar concentration of 0.17 g ml^−1^ of sugar (table 4). We then prepared two types of artificial crop regurgitate both containing 37 mg ml^−1^ bovine serum albumin as a protein source, 0.17 g l^−1^ of sucrose, and 2.5 µl ml^−1^ of formic acid to reach a pH of 3, similar to native crop regurgitate [36]. The fluid used for the juvenile hormone treatment was made by adding a volume of 0.50 µl of a stock solution of juvenile hormone III (CarboSynth, purity 90 area %, concentration 10 µg µl^−1^) in ethanol, to artificial crop regurgitate for a final volume of 60 µl and a final concentration of 83 ng of juvenile hormone per microlitre (0.31 mM). This concentration is the same as the one used in the juvenile-hormone supplemented diet provided to workers in LeBoeuf *et al*. [36], corresponding on average to what is found in the trophallactic fluid of workers [36]. The fluid used for the control treatment included 0.50 µl of pure ethanol instead of juvenile hormone stock solution.

**Table 4.** Sugar and protein concentration of raw crop regurgitate.

colony ID	volume crop regurgitate (µl)	Prot concentration (mg ml^−1^)	sugar concentration (g ml^−1^)
C1	8,5	39.39	0.15
C2	8	27.68	0.16
C3	6,5	66.29	0.23
C4	11	35.72	0.15
C5	8	20.79	0.11
C6	8,8	35.78	0.23

We created two experimental colony fragments, each containing five larvae of about 3 mm long (L3 or bipotent L4 [41]) and five workers. Colony fragments were maintained in individual petri dishes containing one Eppendorf tube filled with water, and another with a 15 g ml^−1^ sugar water solution, both closed with a cotton balls. The five workers included two foragers and three nurses (respectively, individuals found outside of the nest or on the food source, and individuals sitting on the brood pile inside the nest and caring for larvae). For each colony, one of the two colony fragments was assigned to the juvenile hormone treatment and one to the control treatment, resulting in six colony fragments per treatment with a total of 30 larvae (figure 1). The day after colony splitting (day 0), the metabolic rate of larvae from each colony fragment was measured, through measurement of overall O_2_-consumption divided by overall larval mass of each fragment. In addition, each larva was individually weighed (scale: Analytical Series, model FAS124, FisherBrand) and laterally photographed (under binocular with Visicam HDMI6) to confirm the absence of initial differences in body size between the two groups. Forty-eight hours after setting the experimental colony fragments, the handfeeding treatment began (day 1). This consisted of handfeeding each larva three times per week until pupation or death of the larva, with artificial crop regurgitate solution containing juvenile hormone to larvae from the *juvenile hormone treatment,* or without juvenile hormone to larvae from the *control treatment*.

Each handfeeding session consisted of first isolating larvae from their workers and placing them on their back into adapted holes made in a Play-Doh plate. Then using a Hamilton syringe of 10 µl, a 0.5 µl droplet of artificial crop regurgitate solution was placed on the mouth parts of each larva, while using a needle to keep the mouth accessible. An hour later, each larva was cleaned with a moistened cotton-tipped toothpick before being returned to their respective workers. Larvae often clearly drank the fluid (electronic supplementary material, video). In order to better visualize whether larvae had drunk the fluid, the artificial crop regurgitate solution included the blue dye erioglaucine (final concentration 0.1 mg ml^−1^). To minimize fluid transfer from larvae to workers, we made sure that no trace of coloured fluid remained on the mouth parts of larvae before putting them back in contact with workers. To ensure that larvae were hungry before each handfeeding session, and that workers would be well fed when the food larvae were returned to them, workers were provided with artificial diet [58] during the same period when larvae were being fed. In order to monitor how much workers might have fed their larvae, the worker's artificial diet contained 0.03 g l^−1^ of fluorescein (Sigma Aldrich).

To assess worker-to-larvae food transfer, the larvae were photographed on day 22 under UV light and larvae fluorescence was measured. Additionally, each picture included: (i) a negative control consisting of larvae handfed with artificial crop regurgitate containing no fluorescein; and (ii) a positive control consisting of larvae handfed with artificial crop regurgitate containing 0.09 g l^−1^ of fluorescein. The handfeeding of these control larvae was done 1 h 30 min prior to imaging. The analysis of larval brightness was done using GIMP v.2.10.30 software (exposure: 12.616; black level: 0.001), by measuring the average brightness of the larvae, standardized by the larvae area.

Before each handfeeding session larvae were imaged laterally under a stereomicroscope (magnification 30×) for further measurement of larval curvature as a possible indicator of begging behaviour, and the number of larvae and pupae per colony fragment were counted. Each time a pupa was observed it was immediately isolated from the rest of the fragment, to prevent cannibalism by workers and placed into a 96-well plate until pupal measurement. In order to monitor larval growth and changes in metabolic activity over time, individual larval body mass was measured once per week. The metabolic rate of the set of larvae of each colony fragment was measured after 14 days of treatment.

### 4.3. Metabolic rate measurement

O_2_-consumption of the group of larvae from each colony fragment was measured using the MicroRespiration system (UNISENSE Denmark) following their protocol. For each colony, fragment larvae were isolated from their workers and all larvae were placed in a micro-respiration chamber (*v* = 1.28 ml), sealed with 0.5% agar and paraffin oil. The O_2_ micro sensor needle was placed through the chamber lid hole to measure the O_2_-consumption, while a thermosensor was placed next to the glass chamber to measure the temperature. Real-time O_2_-consumption was recorded for 3 min starting 1 min after placing the needle of the micro sensor and viewed using SensorTraceBasic v. 3.3.275. Each set of larvae of each fragment was weighed (scale: Analytical Series, model FAS124, FisherBrand) directly after the O_2_-consumption measurement. We calculated the respiration rate from the O_2_-consumption during the time of measurement (180 s) adjusted for the total mass (mg) of larvae. We calculate ‘metabolic rate’ as the slope of O_2−_consumption (µmol l^−1^) plotted against time (s), divided by ant mass (mg), multiplied by the chamber volume (ml) [59].

### 4.4. Pupal measurement

We measured morphological traits (head width and scape length) of pupae after metamorphosis. The cocoon was removed from late-stage pupae (post metamorphosis). The pupa was placed on its back and photographed for later morphological measurement of head width. As the standardization of the angle for measuring scape length was not possible on the pupae, scape length was photographed and measured on the emerged adult on cut antennae. Measurement was performed with ImageJ (ImageJ bundled with 64-bit Java 8).

### 4.5. Larval individual identity tracking over time

To approximate larval identity over weekly body mass measurements, we defined two algorithms. For one algorithm, we assumed (i) that the ranking of body mass remained constant between consecutive two weeks; (ii) that the heaviest larva was most likely to have pupated, in case of pupation; and (iii) that the lightest larvae is most likely to have died, in case of larval death (i). Because of these assumptions we qualified this algorithm as ‘restrictive’. As this restrictive algorithm could have biased our analyses, we also used another algorithm which we qualified as ‘conservative’. In this algorithm, larval identity across weeks was randomly assigned without the assumption that larvae increase in weight over time, but with two constraints: (i) that larvae cannot grow more than 112.5% between consecutive weeks (corresponding to a growth rate = [mass week*_t_*_+1_/mass week_t_] × 100 = 112.5); and (ii) larvae can only pupate when reaching a minimal weight of 3.8 mg. The choice of a maximal growth rate of 112.5% comes from the observation in our dataset, the maximal growth rate observed was 112.5%, where in colony C2 in the juvenile hormone treatment, the two remaining larvae at day 28 were 4 and 5 mg while they were 8.5 and 8.8 mg at day 35. The choice of 3.8 mg comes from the fact that among the 13 larval mass records for we which we were certain that they were the last mass recorded before pupation, the smallest larva weighed 3.8 mg (observed in colony C3 of the juvenile hormone treatment). In contrast to the restrictive algorithm, the conservative algorithm is highly random. Thus, we created 10 datasets through simulations of larval identity using the conservative algorithm.

### 4.6. Larval begging

To investigate the influence of juvenile hormone supplementation on larval begging, we measured the area between the mouth parts and the rest of larval body on lateral photos of larvae, standardized by the total area of the larva, see the electronic supplementary material, figure S7. We used this measure reflecting the curve of larval neck as a proxy of larval begging.

### 4.7. Statistical analysis

Statistical analyses were conducted in R v. 4.1.2 using the packages *car* and *lme4*. By default, the different models used were implemented with original-colony and colony fragment identity as double random factors. For all linear models used, the normality of the residuals as well as the absence of over- or under-dispersion were systematically verified with a Kolmogorov–Smirnov test and a dispersion test, respectively, using the R package *DHARMa*. Therefore, to comply with model assumptions, the response variables were transformed accordingly. To analyse larval survival and the proportion of larvae that turned into pupae, we ran two survival mixed models using the R package *coxme*(): the first included right-censored number of days until death per individual, the second was the days until pupation per individual, and for both, we implemented the treatment as the response variable. To test the influence of treatment on the morphology of the resulting adults, we ran three different LMMs, each with treatments as explanatory factor and with head width, scape length or the ratio between these variables as the response variable. To test the influence of the treatment on final larval mass, we ran an LMM with the last record of larval body mass before pupation (excluding larvae that died before pupating) as the response variable and treatment as a factor. To test the influence of the treatment on larval growth over time, we ran an LMM with the square root of individual body mass of the larvae that survived until pupation as the response variable and treatment in interaction with time in days as explanatory variables.

To look at the importance of nutrients accumulated over time in relation to juvenile hormone supplementation, we calculated the change in mass of each larva from the start of the experiment until pupation and we ran an LMM with the log-transformation of this variable as response and treatment in interaction with the number of handfeeding sessions as explanatory variables. As for these three last models, the response variables, ‘last mass record before pupation’, ‘individual-larval mass' and ‘Δmass’, depended on our assignment of larval identity, we ran these models on datasets built with the two algorithms, including 10 simulations of the conservative algorithm. To test the influence of the treatment on larval begging behaviour, we ran a binomial generalized linear mixed model with the presence of begging larvae per fragment as the response variable and treatment in interaction with time in days as explanatory variables. For this model, original-colony identity was implemented as single random factor. To compare the larval fluorescence between the juvenile hormone treatment, and the different control treatments at day 22, we ran an LMM with average-larval brightness as the response variable and a variable treatment including the four levels: juvenile hormone treatments, control treatment, negative control fluorescence treatment and positive control fluorescence treatment, as factor; implemented with original-colony, and picture identity as double random factors. However, because for this model there was heterocedasticity of the residuals (see the electronic supplementary material, codes), we used a non-parametric pairwise Wilcoxon rank test to compare the different groups. To look at the effect of the juvenile hormone treatment on larval metabolic rate, we ran a model with overall larval metabolic rate per colony fragment transformed at the power 0.01 as the response variable, treatment in interaction with time as explanatory factors and original-colony identity as a single random factor. In this model, the variable time was categorial with two levels: day 1 and day 14.

## Data Availability

The data are provided in the electronic supplementary material [60].
